# Clinical, Molecular, and Zoonotic Perspectives on Human Cases of *Cryptosporidium* sp. OTUi

**DOI:** 10.3201/eid3205.260128

**Published:** 2026-05

**Authors:** Tine Graakjær Larsen, Edgar Baz-González, Marianne Lebbad, Marielle Babineau, Anson V. Koehler, Lene Nielsen, Christen Rune Stensvold

**Affiliations:** Statens Serum Institut, Copenhagen, Denmark (T.G. Larsen, C.R. Stensvold); Universidad de La Laguna, San Cristóbal de La Laguna, Tenerife, Canary Islands, Spain (E. Baz-González); Sjöbjörnsvägen, Stockholm, Sweden (M. Lebbad); The University of Melbourne, Melbourne, Victoria, Australia (M. Babineau, A.V. Koehler); Copenhagen University Hospital, Herlev and Gentofte, Denmark (L. Nielsen).

**Keywords:** parasites, *Cryptosporidium* OTUi, Apicomplexa, epidemiology, parasitology, protozoa, public health microbiology, surveillance, zoonoses, bats, Australia, Bali, Indonesia, Denmark

## Abstract

We report a case of *Cryptosporidium* sp. OTUi identified in a tourist from Denmark who recently traveled to Indonesia. Previous detections include a traveler from Australia returning from Bali, a bat from the Philippines, and a patient from Australia. On the basis of those findings, we believe zoonotic transmission is plausible.

Surveillance for human cryptosporidiosis cases has been conducted in Denmark since 2023. Clinical microbiology departments can submit original fecal material from positive cases to Statens Serum Institut (SSI; Copenhagen, Denmark) for confirmation and molecular characterization (*1*). We followed that established process for a case of *Cryptosporidium* sp. OTUi identified in a person who had recently traveled to Indonesia.

## The Study

During travel to Bali, Indonesia, a woman from Denmark experienced gastrointestinal symptoms including vomiting and diarrhea. She sought medical care after her return home because symptoms persisted. Multiplex PCR (QIAstat-Dx Gastrointestinal Panel; QIAGEN, https://www.qiagen.com) performed on a fecal sample from the patient at a regional clinical microbiology department identified *Cryptosporidium* spp., Shiga toxin–producing *Escherichia coli*, and norovirus group I and group II.

The woman was interviewed for surveillance purposes and reported having experienced symptoms for ≈14 days. Vomiting developed early, followed by diarrhea. She spent 2 weeks in Bali, primarily in Ubud, with a short stay in Canggu. Exposures included swimming pools and seawater. She reported no cave visits and no direct animal contact, except possible contact with a dog. She observed bats, particularly in Canggu. Similar symptoms developed in 2 travel companions shortly after her illness onset, but their diagnostic status was unknown.

As part of routine *Cryptosporidium* typing at SSI, we performed nested PCR targeting the 60-kDa glycoprotein (*gp60*) gene by using a previously described protocol (*2*). The sequence obtained (GenBank accession no. PX525565; subtype A17) shared >99% identity with another isolate (accession no. KJ506837; subtype A15G1), a relatively short sequence deposited in GenBank as *Cryptosporidium* sp. OTUi subtype AVK-2014. The sequence originated from a woman from Australia who had diarrhea in 2014 after returning from Bali (*3*). Both sequences belong to the same unnamed subtype family, defined as a group of phylogenetically related *gp60* sequences. A third sequence (GenBank accession no. PX092402; subtype A12G1), identified in 2024 in a woman from Australia with unknown travel history, also belonged to the same subtype family (*4*). Each of the 3 sequences represents a distinct subtype, as evidenced by differences in the TCA and TCG configuration within the repetitive region.

**Figure 1:**
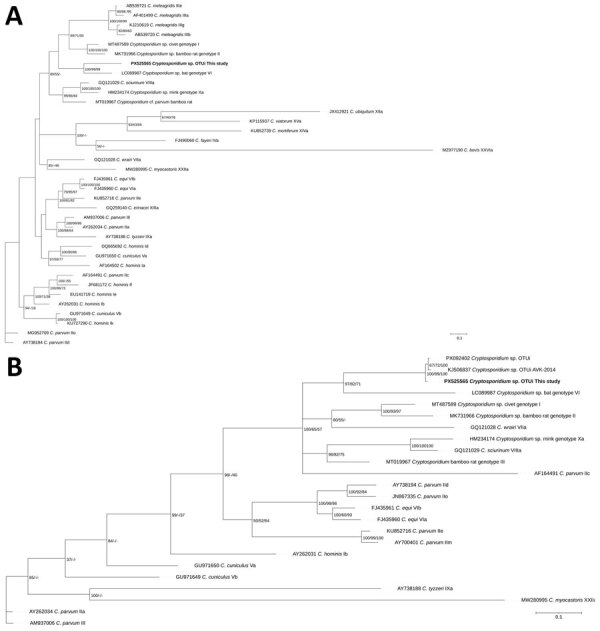
Phylogenetic trees inferred by using Bayesian analysis on the basis of partial nucleotide sequences of the *gp60* gene of *Cryptosporidium* sp. OTUi (GenBank accession no. PX525565) obtained from a woman in Denmark who had traveled to Indonesia (bold text) in a study of the clinical, molecular, and zoonotic perspectives on human cases of *Cryptosporidium* sp. OTUi. A) Analysis including reference sequences (n = 35) retrieved from GenBank. B) Because sequences from previous human cases in Australia (GenBank accession nos. KJ506837 and PX092402) were shorter, we used a reduced alignment, including relevant reference sequences (n = 23). For Bayesian analyses, substitution models were selected on the basis of the lowest Akaike information criterion score, using a general time reversible with invariable site plus discrete gamma model substitution. We performed Bayesian inference by using MrBayes v3.2.7 (*13*) with 5 million generations, 4 chains, and sampling every 1,000 generations. The first 25% of trees were discarded as burn-in. We assessed convergence was assessed by SD of split frequencies <0.01 and a potential scale reduction factor of 1.0. Posterior probabilities and bootstrap support values from maximum-likelihood and neighbor-joining analyses are shown to the right of the nodes. Scale bars indicate substitutions per site.

Because of the limited length of available human *gp60* sequences for comparison, we constructed 2 phylogenetic trees of *Cryptosporidium* sp. *gp60* subtype families by using sequence alignments of different lengths (Figure 1, panels A, B). For the longer alignment (Figure 1, panel A), the ACA and TCA repeat region was removed, but it was kept in the analysis of the shorter fragment (Figure 1, panel B). Our analysis reveals that all 3 human-derived *Cryptosporidium* spp. OTUi sequences cluster together (Figure 1, panel B), and that their closest *gp60* relatives belong to the *Cryptosporidium* spp. bat genotype VI subtype family. Because *gp60* is a highly polymorphic locus used for subtyping and is known to exhibit recombination in some species, the phylogenetic patterns inferred from this marker reflect subtype family relationships but might not reflect species-level relationships.

**Figure 2:**
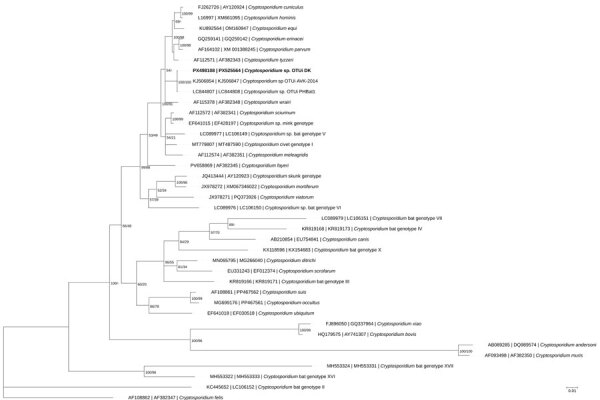
Phylogenetic tree inferred by partitioned Bayesian analysis on the basis of concatenated *ssu* and actin gene sequences of *Cryptosporidium* spp., including *Cryptosporidium* sp. OTUi identified from a woman in Denmark who had traveled to Indonesia (bold text) in a study of the clinical, molecular, and zoonotic perspectives on human cases of *Cryptosporidium*. GenBank accession numbers for *ssu* and actin sequences are provided. Alignments of *ssu* and actin were concatenated according to species identity and OTUi sample identity; only taxa with sequences available for both loci were included. For Bayesian analyses, substitution models were selected on the basis of the lowest Akaike information criterion score (general time reversible with invariable site plus discrete gamma model substitution for *ssu* and actin). Partitioned Bayesian inference was performed by using MrBayes v3.2.7 (*13*) with 10 million generations, 4 chains, and sampling every 1,000 generations; the first 25% of trees were discarded as burn-in. Convergence was confirmed by SD of split frequencies <0.01 and a potential scale reduction factor of 1.0. Posterior probabilities and bootstrap support values from maximum-likelihood analysis are shown to the right of the nodes. Scale bar indicates substitutions per site.

We performed amplification and sequencing of the small subunit rRNA (*ssu*) and actin genes according to established methods (*5*,*6*). The *ssu* sequence obtained from the case in Denmark (GenBank accession no. PX498108) shared >99% identity with OTUi AVK-2014 (accession no. KJ506854) (660/661 bp), the *ssu* sequence derived from the tourist from Australia returning from Bali in 2014; PHBat1 (accession no. LC844807) (674/675 bp), derived from bat feces collected in the Philippines in 2019 (*7*); and the 2024 sample obtained from the woman from Australia (accession no. PV981443) (679/680 bp). The actin sequence from the case in Denmark (GenBank accession no. PX525564) shared >99% similarity with the actin sequence from PHBat1 (accession no. LC844808) (952/954 bp). The phylogenetic tree inferred from Bayesian analyses of concatenated *ssu* and actin sequences (Figure 2) reveals clustering of the 3 OTUi sequences in their own well-supported branch, which is nested within a broader group of branches comprising multiple *Cryptosporidium* spp. and genotypes.

The simultaneous detection of Shiga toxin–producing *E. coli* and norovirus group I and group II is not readily explained by bats as a potential source, because neither pathogen is typically associated with bats (*8*,*9*). Norovirus likely accounted for the acute vomiting period, whereas the prolonged diarrhea is consistent with cryptosporidiosis. Because of the concurrent detection of 3 enteric pathogens and the temporal clustering of gastrointestinal illness (of unknown cause) among the patient’s 2 travel companions, a more plausible explanation is shared exposure to food or water contaminated with fecal material from multiple human or animal sources, rather than transmission from a single reservoir. Contamination of food or water with bat feces is a plausible transmission pathway, particularly where bats roost near agricultural areas and water sources. Similar mechanisms have been proposed for Nipah virus transmission through contaminated food products (*10*,*11*). Ubud and Canggu represent semiurban environments, and human activity overlaps with wildlife habitats, potentially enabling exposure. Also, produce sold within those villages can have originated from more bat-friendly areas. Thus, contamination of water or food with bat feces seems a plausible source of this human *Cryptosporidium* sp. OTUi infection; however, alternative human or animal sources should also be considered.

Bats are typically infected with bat-specific *Cryptosporidium* spp. genotypes, of which >20 have been identified on the basis of *ssu* gene sequencing. The risk of zoonotic transmission from bats is generally considered low because most described bat genotypes are phylogenetically distinct from *Cryptosporidium* spp. commonly found in humans, and they have been detected exclusively in Chiroptera. In addition, only a few instances of human-pathogenic species (*C. parvum*, *C. hominis*, and *C. tyzzeri*) have been detected in bats, without evidence of active infection, suggesting that bats might serve only as mechanical carriers (*12*).

Only 1 *gp60* sequence related to bat genotypes is currently available in GenBank, specifically *Cryptosporidium* spp. bat genotype VI (accession no. LC089987). This scarcity of sequence data might be attributable to primer specificity, because the *gp60* gene exhibits extensive genetic polymorphism or because *gp60* is a single-copy gene, which can affect analytical sensitivity. The primers used in this study amplify a fragment of the *gp60* gene from *C. parvum*, *C. hominis*, and other phylogenetically related species, including *Cryptosporidium* spp. bat genotype VI and *Cryptosporidium* sp. OTUi, but might not amplify other bat genotypes.

The high sequence identity across loci among geographically and temporally distinct human and bat isolates suggests a shared epidemiologic origin. Phylogenetic proximity to *Cryptosporidium* spp. bat genotype VI on the basis of the *gp60* gene further supports a zoonotic link. The association of 2 human cases with travel to Bali suggests this *Cryptosporidium* spp. genotype might be endemic in Bali and potentially maintained by a zoonotic reservoir. The 2019 bat sample originated from a forest roundleaf bat in the Philippines, a nonmigratory species native to the Philippines but not to Bali (*7*). Nevertheless, related bat species inhabit Bali, and connections between bat populations might occur through limited migration or through other hosts, enabling spillover to humans. Subsequent shedding of *Cryptosporidium* sp. OTUi by infected humans could enable transmission through fecal contamination of food or water, amplifying the risk to others.

## Conclusions

This investigation highlights the value of molecular surveillance and multilocus typing for detecting emerging *Cryptosporidium* genotypes and for elucidating potential infection sources. Clustering of *Cryptosporidium* sp. OTUi among travelers returning from Bali, combined with its phylogenetic relationship to a bat-associated genotype, supports regional endemicity and supports the hypothesis of bats being a reservoir for human cryptosporidiosis. Improved genomic characterization of bat-associated *Cryptosporidium* infection and enhanced environmental surveillance are needed to better understand transmission dynamics and assess the public health significance of this parasite.
